# Role of mitochondrial DNA level in epidural-related maternal fever: a single-centre, observational, pilot study

**DOI:** 10.1186/s12884-024-06551-7

**Published:** 2024-05-03

**Authors:** Christina Hafner, Marita Windpassinger, Eva Verena Tretter, Katharina Anna Rebernig, Sophie Marie Reindl, Beatrix Hochreiter, Sabine Dekan, Patrick Haider, Herbert Kiss, Klaus Ulrich Klein, Peter Wohlrab

**Affiliations:** 1 Department of Anaesthesia, Intensive Care Medicine and Pain Medicine, Division of General Anaesthesia and Intensive Care Medicine, Medical University of Vienna, Vienna, Austria; 2 Department of Pathology, Medical University of Vienna, Vienna, Austria; 3 Department of Internal Medicine II, Division of Cardiology, Medical University of Vienna, Vienna, Austria; 4 Department of Obstetrics and Gynaecology, Division of Obstetrics and Feto-Maternal Medicine, Medical University of Vienna, Vienna, Austria; 5 Department of Anaesthesia, Intensive Care Medicine and Pain Medicine, Division of Cardiothoracic and Vascular Anaesthesia and Intensive Care Medicine, Medical University of Vienna, Spitalgasse 23, Vienna, 1090 Austria

**Keywords:** Epidural-related maternal fever, Local anesthetics, Fever, Toxicity

## Abstract

**Introduction:**

Epidural analgesia has been associated with intrapartum maternal fever development. Epidural-related maternal fever (ERMF) is believed to be based on a non-infectious inflammatory reaction. Circulating cell-free mitochondrial deoxyribonucleic acid (mtDNA) is one of the possible triggers of sterile inflammatory processes; however, a connection has not been investigated so far. Therefore, this study aimed to investigate cell-free mtDNA alterations in women in labour with ERMF in comparison with non-febrile women.

**Material and methods:**

A total of 60 women in labour were assessed for maternal temperature every 4 h and blood samples were obtained at the beginning and after delivery. Depending on the analgesia and the development of fever (axillary temperature ≥ 37.5 °C), the women were allocated either to the group of no epidural analgesia (*n* = 17), to epidural analgesia no fever (*n* = 34) or to ERMF (*n* = 9). Circulating cell-free mtDNA was analysed in the maternal plasma for the primary outcome whereas secondary outcomes include the evaluation of inflammatory cytokine release, as well as placental inflammatory signs.

**Results:**

Of the women with epidural analgesia, 20% (*n* = 9) developed ERMF and demonstrated a decrease of circulating mtDNA levels during labour (*p* = 0.04), but a trend towards higher free nuclear DNA. Furthermore, women with maternal pyrexia showed a 1.5 fold increased level of Interleukin-6 during labour. A correlation was found between premature rupture of membranes and ERMF.

**Conclusions:**

The pilot trial revealed an evident obstetric anaesthesia phenomenon of maternal fever due to epidural analgesia in 20% of women in labour, demonstrating counterregulated free mtDNA and nDNA. Further work is urgently required to understand the connections between the ERMF occurrence and circulating cell-free mtDNA as a potential source of sterile inflammation.

**Trial registration:**

NCT0405223 on clinicaltrials.gov (registered on 25/07/2019).

**Supplementary Information:**

The online version contains supplementary material available at 10.1186/s12884-024-06551-7.

## Introduction

Epidural analgesia is commonly used to relieve labour pains, and labour in 20% of women is associated with increased body temperature [1]. The underlying epidural-related maternal fever (ERMF) mechanisms remains unclear although the causal relationship between maternal fever and epidural analgesia is well-known [2, 3].

A current hypothesis is that ERMF is a non-infectious inflammatory reaction that is putatively triggered by damage-associated molecular patterns (DAMPs), which can activate the inflammasome [4]. Interestingly, several in vitro studies demonstrated that local anaesthetics have the potential to trigger immunomodulation and cell injury due to mitochondrial respiratory chain complex inhibitions [5, 6]. Consequently, mitochondrial damage induces the release of large amounts of reactive oxygen species, which promote acute systemic inflammation via pro-inflammatory mediator production [e.g. cytokines, prostaglandin E2, oxidated mitochondrial deoxyribonucleic acid (mtDNA)] [7, 8]. Additionally, mitochondrial stress induces the escape of mtDNA into the extracellular space, which might not be necessarily a passive release, but a specific and regulated process via several possible routes [9].

Studies on various disease states demonstrated that circulating cell-free mtDNA alterations can be measured in peripheral blood, which reflects the degree of mitochondrial injury. Although, for preeclampsia mtDNA dysregulations have been described by different study teams [10, 11], no in vivo study has yet investigated the correlation between cell-free mtDNA alterations and ERMF. Different experimental approaches revealed that local anaesthetics, such as bupivacaine and (to a lesser extent) ropivacaine, have an impact on mitochondrial function, induce ROS generation and are dose-dependently pro-apoptotic [5].

Therefore, this pilot study aimed to investigate alterations of plasma circulating cell-free mtDNA levels in women in labour with ERMF in comparison to women in labour with epidural analgesia an no fever and women without epidural analgesia.

## Material and methods

In this study 105 women participated and 60 were included. The inclusion criteria were women aged ≥ 18 years, ≥ 37 gestational weeks, singleton pregnancy and nullipara or primipara. The exclusion criteria were women aged ≥ 45 years; ≥ 42 gestational weeks; fever within the last 14 days; conversion to caesarean delivery; preeclampsia; haemolysis, elevated liver enzymes and low platelet syndrome; intrauterine growth reduction; gestational diabetes mellitus or autoimmune disease. In total 45 patients were excluded due to conversion to caesarean delivery (*n* = 17) or missing data (temperature measurement or blood samples).

### Study setting

After arrival in labour room (T0), written informed consent was obtained and baseline demographic data (including age, actual weight and gestational weeks), as well as axillary temperature (Thermoval Basic®, Hartmann, Germany; calibration accuracy ± 0.1 °C), were recorded and blood samples were taken (8 ml of serum and 6 ml of ethylenediaminetetraacetic acid (K2EDTA), BD Vacutainer®, Plymouth, UK). Axillary temperature was measured every 4 h until delivery. Immediately after delivery, maternal and placental blood (8 ml of serum, 6 ml of EDTA) were obtained (T1). All blood samples were centrifuged within 2 h of collection at 4 °C and 3000 g for 30 min, and plasma samples were kept at − 80 °C until processed.

After excluding non-eligible patients, 60 women remained for further analysis (Supplemental Fig. 1). Women, who did not request epidural analgesia for spontaneous labour (*n* = 17) represented the control group of ‘no epidural analgesia’ (no EA). No women in this group developed fever. Women with epidural analgesia without an increased temperature of ≥ 37.5 °C were allocated to the group of ‘epidural analgesia without fever’ (EA no fever, *n* = 34). According to previous studies women with epidural analgesia and a temperature of ≥ 38 °C or 2 consecutive temperatures ≥ 37.5 °C after the onset of labour were assigned to the group of ‘epidural-related maternal fever’ (EA with fever) (*n* = 9) [12–14].

Epidural analgesia was administered to 45 women using a standardized method. After an epidural catheter placement in the lumbar space, 0.2% ropivacaine was titrated to a bilateral sensory level at thoracal 10. A continuous epidural infusion of 0.2% ropivacaine with fentanyl at 2 µg/mL was infused at 8–10 ml/h to maintain the sensory level at thoracal 10.

### Primary outcome

#### Circulating cell-free mtDNA

The DNA was extracted from platelet-poor plasma using Qiagen DNeasy Blood Mini Kit (Qiagen, Venlo, Netherlands) for the circulating cell-free mtDNA analysis, and equal amounts of DNA were analysed via real-time quantitative polymerase chain reaction (qRT-PCR) on a RotorGene Q (Qiagen, Venlo, NL) using primers for the mitochondrial NADH dehydrogenase subunit 2 (ND2) (forward primer: CCCTTACCACGCTACTCCTA; reverse primer: GGCGGGAGAAGTAGATTGAA) and Perfecta SYBR green Fast Mix (Quanta Biosciences, Gaithersburg, USA). The qPCR temperature programme comprised denaturation for 30 s at 95 °C and 42 cycles for 5 s at 95 °C, 30 s at 62 °C and 20 s at 72 °C.

### Secondary outcome

#### Circulating cell-free nuclear DNA (nDNA)

Plasma-extracted DNA was further analysed for nuclear DNA using primers for ß-actin (forward primer: AGACGCAGGATGGCATGGG; reverse primer: GAGACCTTCAACACCCCAGCC) and the same temperature settings as for ND2 during qPCR. The qPCR output data from mtDNA and nDNA quantifications are shown in Figures with absolute cyclic threshold (Ct) values. Increased Ct values reflect lower amounts of mtDNA or nDNA.

#### Oxidative damage of cell-free mtDNA

Purine nucleotide (8-OHdG) oxidation in mtDNA was assessed by determining the cyclic threshold (Ct) after treating DNA samples with the DNA glycosylase Fpg, an enzyme that lyses damaged purines creating a nucleotide DNA gap and thereby reducing amplification efficiency (read-out: Ct: enzyme-treated minus Ct enzyme-non-treated samples), in analogy to the previously described method [15].

#### Cytokines and prostaglandin E2

The inflammatory response was measured using enzyme-linked immunosorbent assays for Interleukin (IL)-1ß, IL-6, IL-8 (DuoSet, R&D Systems; Minneapolis, Minnesota, USA) and prostaglandin E2 (PGE2) (Enzo Life Sciences; Lausen, Switzerland) following the manufacturer’s protocol in the maternal plasma at the start of epidural analgesia and delivery, as well as in chord serum after delivery. PGE2 was only analysed if delivery was not induced with prostaglandin.

#### Pathohistological placental examination

A total of 47 placentae were examined by a senior pathologist according to routine protocol, and inflammation was categorised as none, mild (amnionitis), moderate (chorioamnionitis) or severe (funiculitis and/or chorionic vasculitis) [16].

#### Sample size calculation

This study was planned as a pilot study. Due to missing data in this field no formal sample size calculation could be performed. However, based on previous experimental in-vitro data it was hypothesized that a minimum of 8 samples of women with ERMF would be required for mtDNA analysis to detect alterations of circulating cell-free mtDNA in patients with ERMF in comparison to women without EA and women with EA no fever [5]. This sample size is similar to a study, which investigated inflammatory parameters in ERMF [17]. It was expected that ERMF will occur in approximately 20% of women in labour with epidural analgesia. These considerations resulted in a sample size of 40 women in labour with epidural analgesia. The control group (labouring women without epidural analgesia) were expected to require 15 patients.

### Statistical analysis

Statistical analysis was performed using Prism 9.0 software (GraphPad, San Diego, CA, USA). Demographic data are presented as mean ± standard deviation or median (25th–75th percentile). The normality of distribution was assessed using the Kolmogorov–Smirnov test. As indicated, differences of mtDNA levels and cytokineswithin the group were analysed with a paired *t* test and between the groups with one-way analysis of variance (with post hoc testing). Differences between frequencies were calculated using the chi-square test. A *p* value of ≤ 0.05 was considered significant. The Wilcoxon test was used for differences within groups.

### Ethics statement

This pilot study was performed at the Department of Anaesthesia and General Intensive Care at the Medical University of Vienna from July 2019 to December 2020. Ethical approval (Number: 2119/2017) was obtained by the Ethics Committee of the Medical University of Vienna (Chairperson Prof. E. Singer). The study conformed to the Declaration of Helsinki guidelines regarding research on human subjects and followed the tenets of Good Clinical Practice. Written informed consent was obtained from all subjects participating in the trial. The trial was registered before enrolment at clinicaltrials.gov by the principal investigator Klaus Ulrich Klein (NCT04045223 registered on 25/07/2019).

## Results

### Characteristics of women in labour

Table 1 shows the demographic and labour-specific data of women in labour. No significant differences were found between all groups in demographic data (age, weight), routine inflammatory blood parameters (C-reactive protein, leukocytes, lymphocytes, monocytes) and child-specific data (APGAR score, umbilical vein pH, birth weight). Labour duration was significantly longer in women with epidural analgesia (*p* < 0.01) but not in women who developed a fever. Premature rupture of membranes (PROM) was more often present in women with fever (55%) compared to parturients with epidural analgesia without fever (21%) and women without epidural analgesia (6%).

**Table 1:** Demographic, routine inflammatory parameters and labour-specific data. Data are presented as mean (standard deviation), median (25th and 75th percentile) or absolute numbers (percentage)

	**No EA** **(*n* = 17)**	**EA no fever** **(*n* = 34)**	**EA with fever** **(*n* = 9)**
**Demographic data**			
Age (years)	29 (24–32)	29 (26–33)	27 (23–33)
Weight (kg)	80 (19)	78 (16)	74 (13)
**Routine inflammatory parameters**			
C-reactive protein (mg/dl)	0.46 (0.25–0.89)	0.58 (0.31–0.92)	0.38 (0.21–1.00)
Leukocytes (G/l)	12.03 (10.26–14.82)	11.90 (10.28–13.40)	11.90 (8.98–12.90)
Lymphocytes (G/l)	1.8 (1.5–2.0)	1.8 (1.3–2.1)	2.1 (1.5–2.2)
Monocytes (G/l)	0.6 (0.5–0.7)	0.6 (0.5–0.8)	0.7 (0.6–0.8)
Neutrophils (G/l)	8.2 (7.5–10.1)	8.1 (6.9–10.3)	8.1 (6.8–9.3)
**Labour-specific data**			
Para 0	8	29	9
Para 1	9	5	0
Gestation (weeks)	39 (1)	39 (2)	40 (1)
Premature rupture of membrane (N, %)	1 (6%)	7 (21%)	5 (55%)
Prostaglandin-induced labours (N, %)	1 (6%)	7 (21%)	2 (22%)
Labour duration (min)	393 (191–552)	793 (483–1474)	659 (364–886)
Duration of epidural analgesia (min)	N/A	430 (312–652)	491 (449–513)
**Child-specific data**			
APGAR 1 min	9 (9–9)	9 (8–9)	9 (8.5–9)
APGAR 5 min	10 (10–10)	10 (9–10)	10 (9,5–10)
APGAR 10 min	10 (10–10)	10 (10–10)	10 (10–10)
Umbilical vein pH	7.21 (7.16–7.25)	7.23 (7.13–7.19)	7.24 (7.15–7.36)
Birth weight (kg)	3.487 (0.503)	3.319 (0.428)	3.439 (0.438)

### Maternal temperature

All women were afebrile and without clinical signs of infection at the beginning of the study (Table 2). A significantly increased temperature was detected in subjects with ERMF 8 h after the start of the study. ERMF (37.7 °C ± 0.3 °C) developed in 20% of the women who received epidural analgesia. None of the women in the other groups had an increased temperature of > 37.5 °C.

**Table 2:** Maternal temperature at the beginning, 4 and 8 h after the start and inflammatory laboratory parameter at the start of labour. Data are presented as mean (standard deviation)

	**No EA** **(*n* = 17)**	**EA no fever** **(*n* = 34)**	**EA with fever** **(*n* = 9)**
**Maternal temperature**			
Temperature at start (°C)	36.1 (0.5)	36.1 (0.6)	36.4 (0.5)
Temperature 4 h after start (°C)	36.0 (0.7)	36.3 (0.5)	36.3 (1.0)
Temperature 8 h after start (°C)	36.5 (0.6)	36.5 (0.4)	36.9 (0.3)
Temperature at delivery (°C)	36.4 (0.6)	36.6 (0.5)	37.7 (0.3)

### Primary outcome

**Fig. 1:**
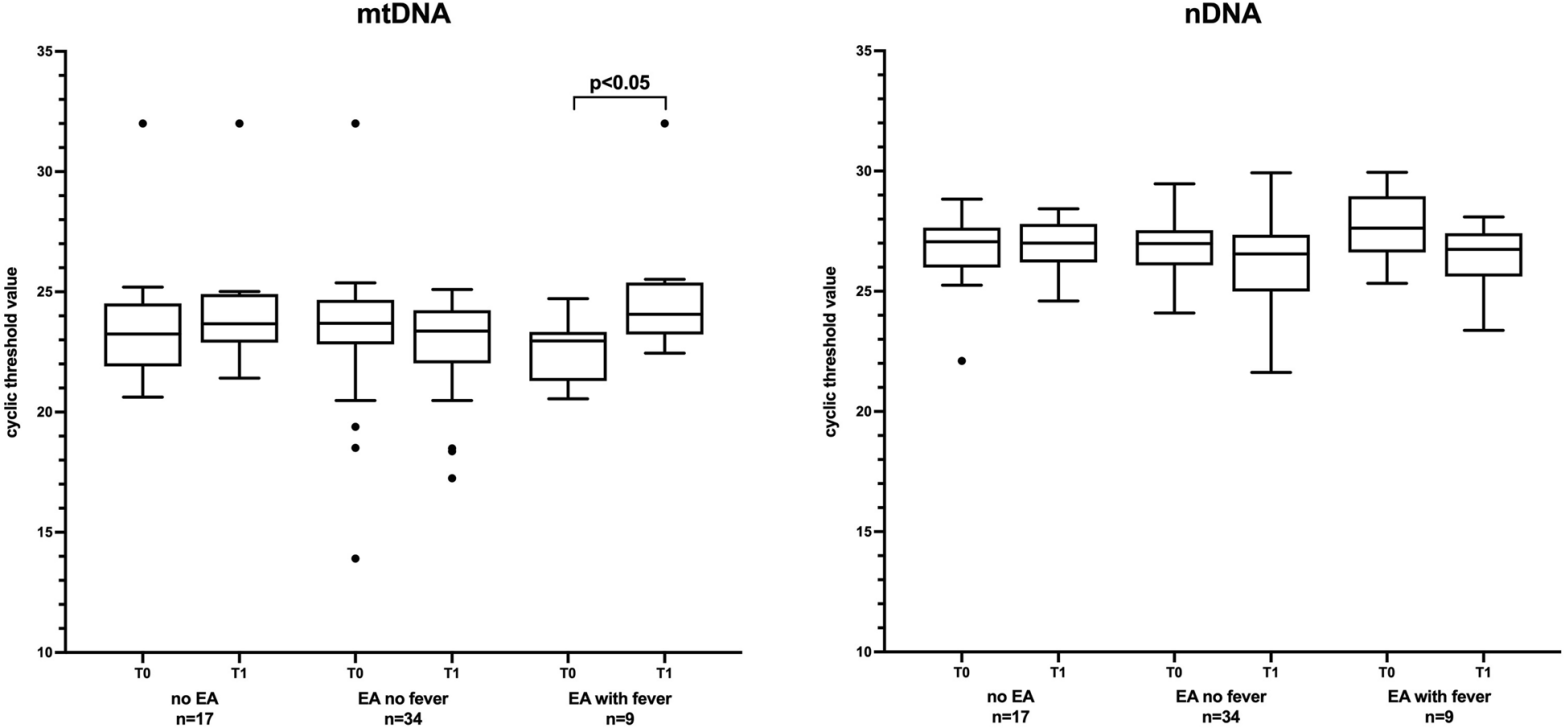
Quantitive real-time PCR assessing cell-free mtDNA and nDNA in maternal plasma at time points T0 (start of epidural analgesia) and T1 (after delivery). qPCR output data of mtDNA and nDNA quantifications are shown as absolute cyclic threshold (Ct) values (median, 25th and 75th percentile; *p < 0.05). Increased Ct values reflect lower amounts of mtDNA or nDNA

Figure 1 shows that women with ERMF demonstrated a greater difference (*p* = 0.04) in the median cyclic threshold value of mtDNA between the two time points, 22.96 (21.29–23.34) vs. 24.07 (23.23–25.39). No significant difference was found within other and between groups. Additionally, no significant difference was found in the median Ct value of cell-free nDNA, although a downward trend was observed in the ERMF group.

Moreover, the degree of oxidative modifications of the cell-free DNA in all samples by a qPCR-based method revealed a higher degree of variation at T0 in women with ERMF (data not shown). However, the statistical evaluation showed no significant difference at T0 between women with ERMF and women with EA without fever (*p* = 0.22).

### Secondary outcomes

#### Inflammatory cytokines

**Fig. 2:**
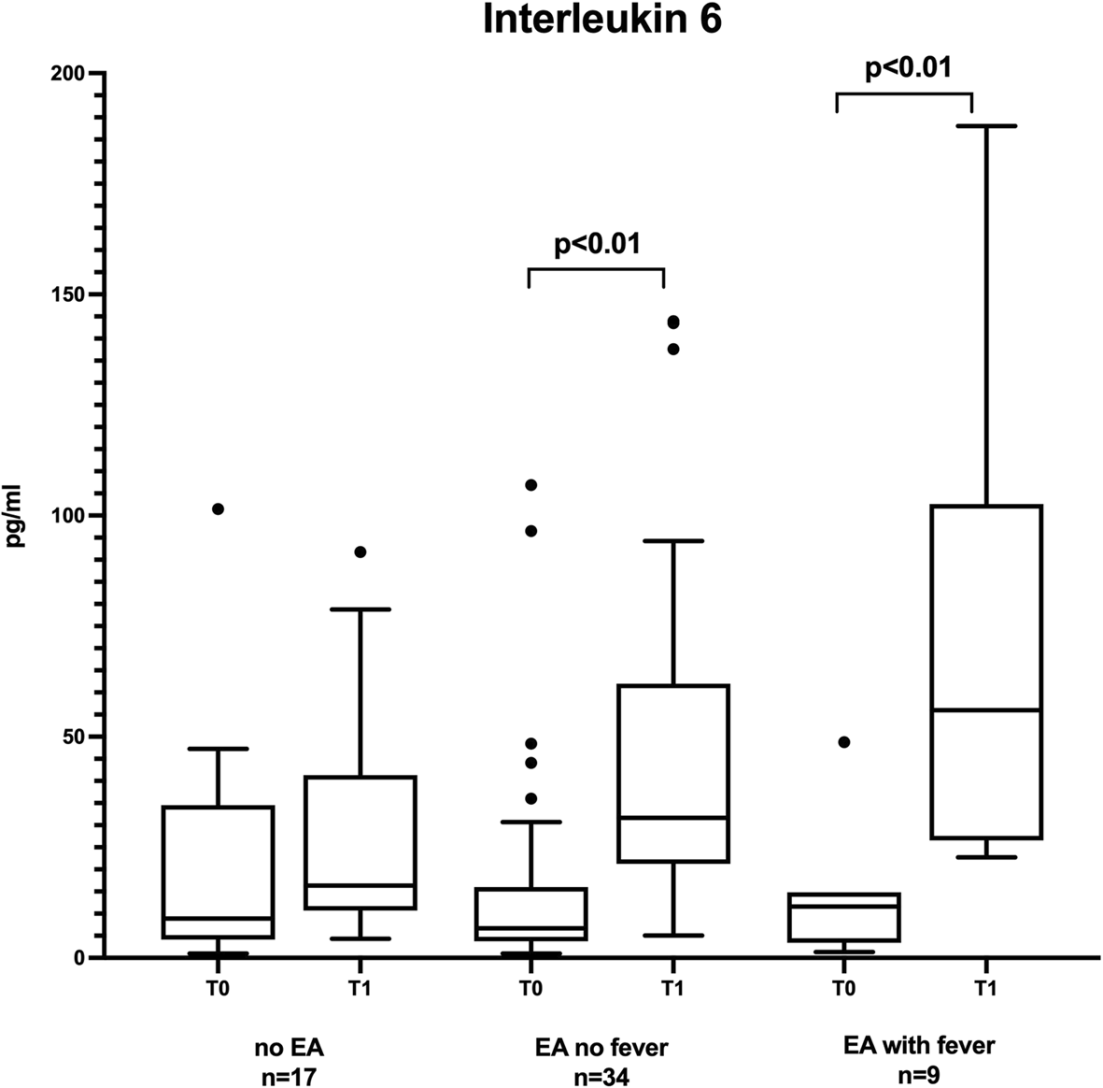
Levels of Interleukin-6 in maternal plasma (at the start and after delivery). Data are presented as median, 25th and 75th percentile. *p* < 0.05 was considered significant

A significant release of IL-6 (Fig. 2) was present in the maternal plasma in women with epidural analgesia (*p* < 0.01), as well as in ERMF (*p* < 0.01) at delivery. Other inflammatory cytokines, such as IL-1ß, IL-8 and PGE2, demonstrated no significant changes within and between the groups (Supplemental Table 1).

#### Placental inflammation

**Fig. 3:**
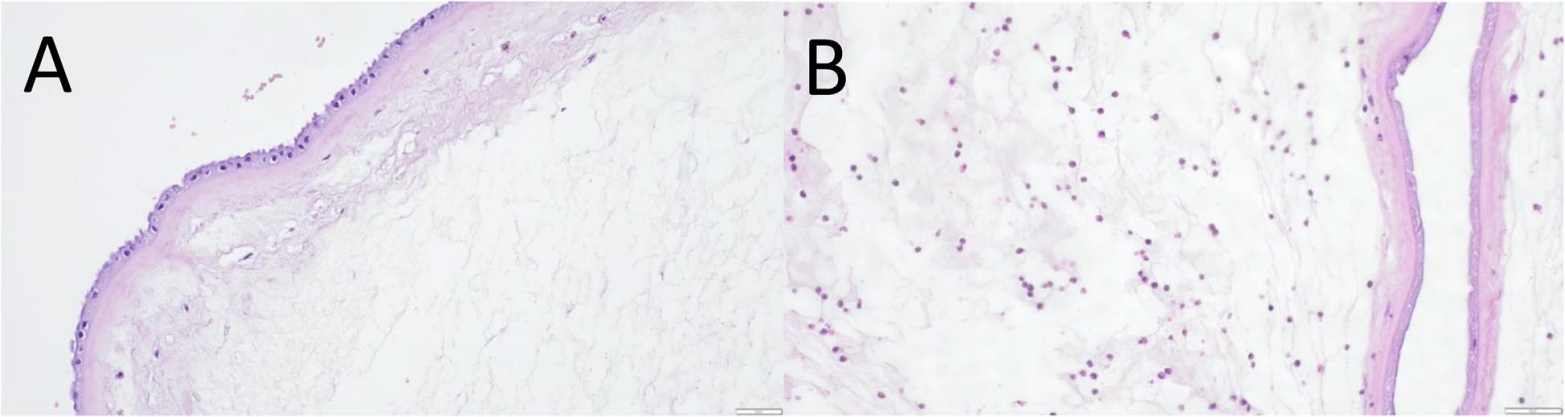
Haematoxylin/Eosin (HE)-staining of amnion. **A** HE-staining of amnion without inflammatory signs. **B** HE-staining of amnion with mild inflammatory signs

Pathohistological placental examination (Fig. 3) showed mild signs of inflammation in 44% of women without epidural analgesia, 59% of women with epidural analgesia and 67% of ERMF cases. Only one incident of moderate placental inflammation was detected in a woman who had epidural analgesia but no signs of a fever (Table 3).

**Table 3:** Pathohistological examination of amnion. Data are presented as absolute numbers (percentage)

	**No EA** **(*n* = 17)**	**EA no fever** **(*n* = 34)**	**EA with fever** **(*n* = 9)**
**Maternal temperature**			
Temperature at start (°C)	36.1 (0.5)	36.1 (0.6)	36.4 (0.5)
Temperature 4 h after start (°C)	36.0 (0.7)	36.3 (0.5)	36.3 (1.0)
Temperature 8 h after start (°C)	36.5 (0.6)	36.5 (0.4)	36.9 (0.3)
Temperature at delivery (°C)	36.4 (0.6)	36.6 (0.5)	37.7 (0.3)

## Discussion

This pilot study was conceived in preparation for further clinical studies, and the results demonstrate that women with ERMF during labour exhibit altered mtDNA dynamics. Furthermore, plasma IL-6 levels significantly increased during labourin women with an epidural catheter.

Confirming previous studies, 20% of women with epidural analgesia developed a temperature increase of > 37.5 °C during labour in this pilot trial [4, 18]. The intrapartum temperature elevation started within 4–8 h after epidural placement. Our study did not reveal an association with a higher body mass index or labour duration as previously reported [18]. However, PROM was associated with ERMF as several studies previously indicated [19]. IL-6 level elevation in plasma from women with epidural analgesia was also previously found in a study by Riley et al. [20].

The course of physiological and molecular events that lead to ERMF remained controversial; however, recent data indicate that maternal pyrexia in the absence of infection involves sterile inflammation processes and/or altered thermoregulation [1, 20]. Several clinical and experimental studies have provided evidence of possible inflammatory mechanisms, including the direct effects on inflammatory cytokine release [17], anaesthetic-induced mitochondrial disturbances, reactive oxygen specie generations and pro-apoptotic developments [5].

The release of mtDNA from mitochondria into the cytoplasm and further into the extracellular space is proposed to occur via different mechanisms, which is not understood in every detail [21]. Cell-free mtDNA levels were increased towards term during healthy pregnancy and returned to initial lower levels 6–8 several weeks after giving birth [22]. However, mtDNA dysregulations have been described during pregnancy in women with preeclampsia resulting in different profiles reported by different study teams, as well as transitional suppressed mtDNA levels [23]. Different experimental approaches revealed that local anaesthetics, such as bupivacaine and (to a lesser extent) ropivacaine, have an impact on mitochondrial function, induce ROS generation and are dose-dependently pro-apoptotic [5]. These findings have prompted us to investigate if secreted mtDNA could be a contributor to inflammatory processes that lead to ERMF. Interestingly, this pilot trial did not reveal increased plasma mtDNA levels in women with ERMF. However, patients with ERMF showed mtDNA dysregulation compared with women without ERMF, who had a stable situation of circulating cell-free mtDNA, during labour. The ERMF group exhibited a decreased circulating cell-free mtDNA during labour, which was statistically significant, and a (non-significant) tendency to higher cell-free nDNA levels. The emerging pattern is similar to what was observed by Cushen et al. in women with preeclampsia, although to a lesser extent, which might probably be due to the lower number of women in our ERMF group [23]. Our study revealed a higher amount of oxidated cell-free mtDNA in the ERMF group at T0; however, it did not reach statistical significance due to the large variation of individual values.

The results of this pilot trial cannot support the theory that ERMF may be explicitly traced back to a local anaesthetic-induced mitochondrial damage and concomitantly altered mtDNA release dynamic; however, the findings demonstrate the importance of future investigations in the field of circulating cell-free mtDNA and sterile inflammation processes in the context obstetric anaesthesiology.

This pilot study was performed in preparation of further clinical studies. One finding in this study was that the transfer from bench to bedside needs more evidence. Due to challenging recruitment of patients with ERMF following clinical studies would benefit from multicenter studies. Furthermore, due to the fact that the timepoints for the determination of mtDNA alterations even aspects during pregnancy should be considered and moreover, non pregnant patients with an epidural analgesia and even patients with a caesaran section should even be observed as control groups.

A major limitation is that due to missing data in this research field no formal sample size calculation could be possible. The defined sample size for for the required women with ERMF in this pilot study were based on a similar work. However, the present data of this pilot study can serve as a basis for designing future clinical trials in this area. Furthermore, limitations include the small sample size besides diversity and ethnicity. Moreover, blood samples were only taken after admission to the delivery room and immediately after birth. Circulating cell-free mtDNA levels during pregnancy and labour progress would be of major interest. In addition a limitation of the method is that only non-hemolytic samples can be analysed. Due to this fact mtDNA levels could not be measured in placental blood.

Importantly, epidural analgesia for women in labour was given as a ropivacaine/fentanyl combination in this pilot study, whereby fentanyl could be a confounding factor concerning the study outcome. Previous studies revealed that fentanyl has an impact on the mitochondria [24] and can decrease the mitochondrial copy number in blood although only in male and not in female mice [25]. However, the significantly decreased cell-free mtDNA only in the ERMF group speaks against this obstacle, because the control group of ‘EA no fever’ that received the anaesthetic combination did not show the effect.

The use of Ct-values to compare groups might appear to the reader as if authors have not further processed their raw data in order to perform calculations of "fold change" relative to the control group. However, this decision was made for the following reason: circulating cell-free nuclear DNA and mtDNA are most probably released from cells independently by different mechanisms. Cell-free nuclear DNA is released from cells undergoing cell death (apoptosis), while mtDNA can be released in other context (cell stress or other triggers). An indicator, that this might also be the case in this study, is shown by the fact, that in the ERMF group the mtDNA shows a downward trend between the different time points, while cf nDNA shows an upward trend. Therefore normalizing cf mtDNA levels to cf nDNA would induce a bias. As we have used the same sample size in all cases and have processed all samples according to the same protocol, we decided to use the raw Ct values to compare the data. This procedure makes the differential effect on cf nDNA and cf mtDNA more visible.

## Conclusion

Up to 20% of women in labour with epidural analgesia might be affected by maternal fever; however, the underlying cause of ERMF is not fully understood. The present study demonstrates cell-free mtDNA dysregulation in women with maternal fever. The results support the theory of sterile inflammation in ERMF and support the urgent need for future investigations, in vivo and in vitro, to elucidate the origin of the ERMF phenomenon.

### Supplementary Information

**Supplementary Material 1.**

**Supplementary Material 2.**

## Data Availability

The datasets used and/or analysed during the current study available from the corresponding author on reasonable request.

## Abbreviations

DAMP: Damage-associated molecular patterns
ERMF: Epidural-related maternal fever
PROM: Premature rupture of membranes
