# Supplementary figures and images for: Role of mitochondrial DNA level in epidural-related maternal fever: a single-centre, observational, pilot study
Source: BMC Pregnancy Childbirth. 2024 May 3;24:341. doi: 10.1186/s12884-024-06551-7 (PMC11067090; doi:10.1186/s12884-024-06551-7)

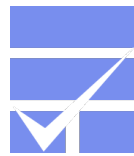

# CONSORT

TRANSPARENT REPORTING of TRIALS

## CONSORT 2010 Flow Diagram

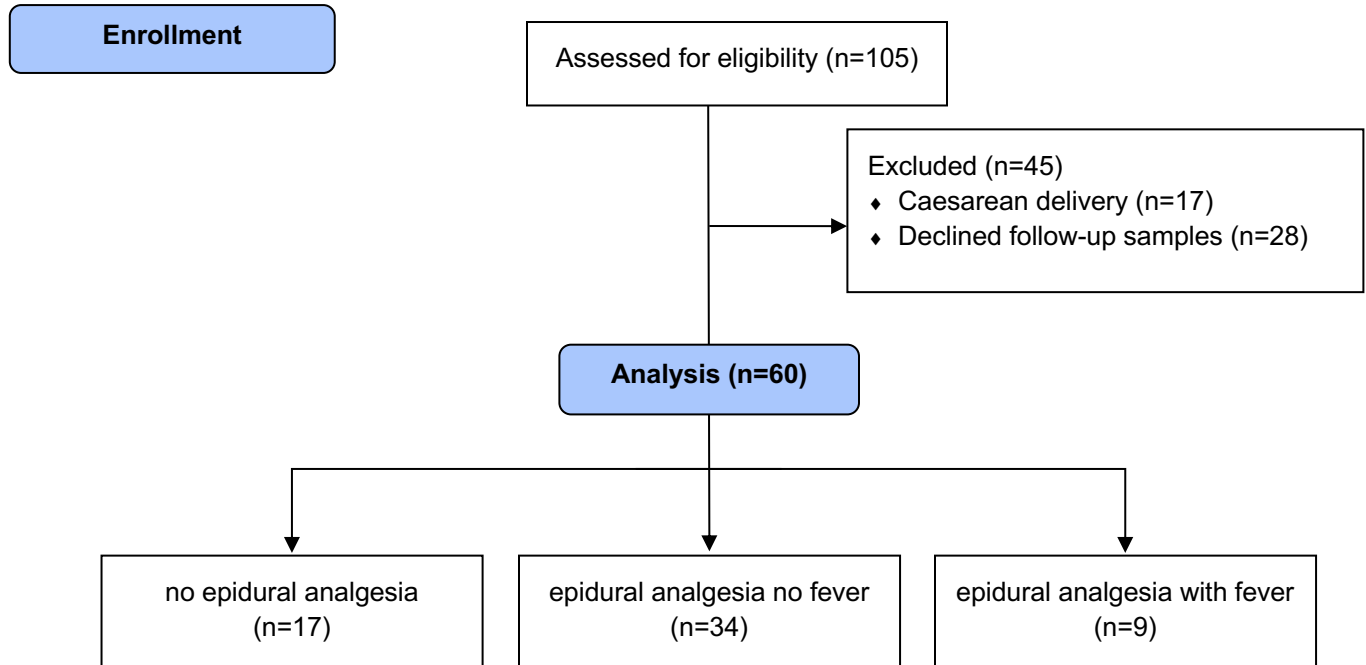

Supplement: Supplementary file 1 — Supplementary Material 1. [file 12884_2024_6551_MOESM1_ESM.pdf]
