# Supplementary material for: Role of mitochondrial DNA level in epidural-related maternal fever: a single-centre, observational, pilot study
Source: BMC Pregnancy Childbirth. 2024 May 3;24:341. doi: 10.1186/s12884-024-06551-7 (PMC11067090; doi:10.1186/s12884-024-06551-7)
Supplement: Supplementary file 2 — Supplementary Material 2. [file 12884_2024_6551_MOESM2_ESM.docx]

|  | **no EA** | **EA** | **EMRF** |
| --- | --- | --- | --- |
| IL-1ß maternal serum at start (pg/ml) | 2.5 (1.7-7.5) | 2.0 (1.2-4.9) | 1.8 (0.9-3.6) |
| IL-1ß maternal serum at delivery (pg/ml) | 1.0 (0.5-6.6) | 1.2 (0.5-5.1) | 1.1 (0.5-1.8) |
| IL-8 maternal serum at start (pg/ml) | 8.1 (5.3-15.4) | 7.9 (6.0-13.8) | 9.6 (4.6-14.7) |
| IL-8 maternal serum at delivery (pg/ml) | 8.4 (3.8-14.5) | 15.8 (8.8-20.5) | 14.55 (6.8-24.8) |
| PGE2 maternal serum at start (pg/ml) | 740 (375-1338) | 970 (580-1835) | 1115 (440-1320) |
| PGE2 maternal at delivery (pg/ml) | 1148 (535-1865) | 785 (320-2750) | 1310 (565-2140) |

**Supplemental Table 1: IL-1ß, IL-8, PGE2**. Data are presented as median (25^th^ and 75^th^ percentile)
